# First Insights into the Genome Sequence of the Alkaliphilic Thermotolerant Bacterium *Clostridium thermoalcaliphilum* JW/YL23-2^T^

**DOI:** 10.1128/genomeA.00368-17

**Published:** 2017-05-18

**Authors:** Anja Poehlein, Alexandra Berg, Gina Welsing, Rolf Daniel

**Affiliations:** a Genomic and Applied Microbiology & Göttingen Genomics Laboratory, Institute of Microbiology and Genetics, Georg-August University of Göttingen, Göttingen, Germany; b Members of the Applied Bioinformatics in Microbiology Course of the Microbiology and Biochemistry M.Sc./Ph.D. program, Georg-August University of Göttingen, Göttingen, Germany

## Abstract

*Clostridium thermoalcaliphilum* is an obligate anaerobic and rod-shaped bacterium isolated from sewage sludge. It is an alkaliphilic thermotolerant organism and utilizes sucrose, glucose, fructose, maltose, cellobiose, amino acids, and Casamino Acids as substrates. The draft genome comprises 2.031 Mbp and 2,027 predicted protein-coding genes.

## GENOME ANNOUNCEMENT

*Clostridium thermoalcaliphilum* is rod-shaped and motile, with 2 to 12 peritrichous flagella. It was isolated from the Atlanta municipal sewage plant (Atlanta, GA, USA) together with its close relative, *Clostridium paradoxum*. A 16S rRNA-based analysis revealed a 2% evolutionary distance between the two species (1).

Genomic DNA of *C. thermoalcaliphilum* JW/YL23-2^T^ was isolated using the MasterPure complete DNA purification kit, as recommended by the supplier (Epicentre, Madison, WI, USA). Illumina paired-end sequencing libraries were generated from the extracted DNA according to the protocol of the manufacturer (Illumina, San Diego, CA, USA). Sequencing was performed with a MiSeq instrument and MiSeq reagent kit version 3, as recommended by the manufacturer (Illumina). Sequencing resulted in 2,691,200 paired-end reads that were trimmed using Trimmomatic version 0.36 (2). Genome assembly with SPAdes version 3.10.0 (3) resulted in 51 contigs (>500 bp) and an average coverage of 272×. For validation of the assembly, Qualimap version 2.1 was used (4). The size of the draft genome and the G+C content were 2.031 Mb and 30.99%, respectively. Compared to other clostridial species, the genome size of *C. thermoalcaliphilum* is relatively small. The software tool Prokka (5) was used for automatic gene prediction and automatic annotation. The draft genome contains 12 rRNA genes, 63 tRNA genes, 1 transfer-messenger RNA (tmRNA) gene, 1,563 protein-coding genes with predicted function, and 464 genes encoding hypothetical proteins. The genome of *C. thermoalcaliphilum* harbors all necessary genes coding for proteins involved in the degradation of glycine and betaine. The corresponding gene cluster shows similarity to the corresponding ones identified in *Sporomusa ovata* (6), *Peptoclostridium acidaminophilum* (7), and* Clostridium tepidiprofundi* (8). We could also identify a proline reductase gene cluster exhibiting structural similarity to the cluster found in *P. litorale* (9). Moreover, we detected all genes (*sel*ABCD), including the tRNA, necessary for incorporation of selenocysteine into proteins (10). In addition, a putative Na^+^-translocating F-type ATPase was present in the *C. thermoalcaliphilum* genome. In *Clostridium paradoxum*, a homologue of this F-type ATPase was analyzed. It was shown that this enzyme functions strictly as a Na^+^ exporter to establish an electrochemical gradient for driving cellular processes (11). Further, we detected that a ferredoxin-NAD^+^ oxidoreductase (Rnf) is encoded by the genome of *C. thermoalcaliphilum*. This enzyme is putatively involved in sodium efflux (11, 12). *C. thermoalcaliphilum* utilizes sucrose, glucose, fructose, maltose, cellobiose, and Casamino Acids as substrates. The fermentation products comprise the acids acetate and lactate (1). Correspondingly, putative genes coding for acetate kinase, phosphate acetyltransferase, and l-lactate dehydrogenase were present in the genome of *C. thermoalcaliphilum*.

### Accession number(s).

This whole-genome shotgun project has been deposited at DDBJ/EMBL/GenBank under the accession no. MZGW00000000. The version described here is version MZGW01000000.
